# Rapid establishment of a HEK 293 cell line expressing FVIII-BDD using AAV site-specific integration plasmids

**DOI:** 10.1186/1756-0500-7-626

**Published:** 2014-09-10

**Authors:** Xiaomei Liu, Han Ping, Chun Zhang

**Affiliations:** Suzhou Municipal Key Laboratory of Molecular Diagnostics and Therapeutics, Suzhou Institute of Biomedical Engineering and Technology, Chinese Academy of Sciences, NO. 88 Keling Road, Suzhou New District, Suzhou 215163 P. R. China

**Keywords:** B-domain-deleted FVIII (FVIII-BDD), Site-specific integration, HEK 293 cell line, Adeno-associated virus (AAV)

## Abstract

**Background:**

Stable human cell lines have gradually become the preferred system for large scale production of recombinant proteins for clinical applications because of their capacity of proper protein post-translational modification and low immunogenicity. However, human cell line development technologies are commonly based on random genome integration of protein expressing genes. It is required to screen large numbers of cell clones to identify stable high producer cell clones and the cell line development process usually takes 6 to 12 months. Adeno-associated virus type 2 (AAV2) Rep protein is known to induce rAAV DNA integration into a specific site (AAVS1) of the human chromosome 19 and integrated transgenes can stably express proteins. We take advantage of this AAV unique feature to develop a rapid protocol to clone a stable recombinant protein expression human cell line.

**Findings:**

We have constructed two plasmids. One plasmid, pSVAV2, contains the AAV rep gene for the synthesis of integrase; the second plasmid, pTRP5GFPFVIII-BDD, contains B-domain-deleted factor VIII (FVIII-BDD) and GFP gene flanked by AAV ITRs. Human embryonic kidney (HEK) 293 cells were co-transfected with the two plasmids and the cells were screened by green fluorescence to establish the recombinant FVIII-BDD cell line. PCR analysis showed that the FVIII-BDD gene has been integrated into the AAVS1 site of human chromosome 19. The FVIII-BDD protein secreted into the extracellular media exhibited coagulant activity.

**Conclusion:**

We developed a method of rapid establishment of human HEK 293 cell line expressing recombinant FVIII-BDD protein with AAV site-specific integration plasmids.

## Findings

### Introduction

In recent years, the number of recombinant proteins used for therapeutic applications has increased dramatically. Stable human cell lines have been extensively used as a preferred expression system for recombinant proteins because their biochemical machineries are capable of carrying out most of the post-translational folding and processing required for generating functional and mature protein and provide high protein productivity [1]. While traditional stable transfection strategies typically involve the random integration of foreign genes into chromosomes, it is difficult to obtain homogenous level of protein expression among individual transfected cells, and the gene amplification process results in large genomic rearrangements that lead to further heterogeneity in protein expression levels. Furthermore, the production cell line development process typically requires 6 to 12 months [2]. Site specific integration of protein expressing genes offers an alternative strategy to develop high producing and stable clones in a predictable manner [3]. This greatly improves the recombination efficiency through the use of recombinases, in contrast to the traditional homologous recombination.

Adeno-associated virus (AAV) is the only known eukaryotic virus which has the capability of undergoing site-specific integration into the human genome. Wild-type (wt) viral DNA recombines at chromosome (ch) -19.13.3 qter at a frequency of 70% [4, 5]. Some elements have been involved in site-specific integration: the AAV Rep68/78 protein in trans as recombinase [6], and the AAV inverted terminal repeat (ITR) in cis (this may not be essential if the P5 promoter, which contains a Rep-binding site [RBS], is present), and AAVS1, a unique sequence in the human genome [5, 7–9]. Rep-dependent integration of ITR-flanked cassettes has been reported in human primary cells and human cell lines. The majority of integrations were site specific and occurred within a 1000-bp region of AAVS1 [10].

Hemophilia A or factor VIII deficiency is a common X-linked genetic bleeding disorder in humans, occurring in about 10-20 males per million [11]. Recombinant FVIII (rhFVIII) products have been available for 20 years; so far, all are produced in non-human cell lines, which produce recombinant proteins with a non-human pattern of post-translational modifications (PTMs) [12]. Recently, human cell lines have been used to produce rhFVIII and represent an improvement in the category of rhFVIII production [12–15]. Both the avoidance of non-human glycan structures and the achievement of complete sulfation are proposed to lessen the intrinsic immunogenicity of human-derived rhFVIII compared with current rFVIII products.

In this study, we have constructed two plasmids. One plasmid, pSVAV2, contains the AAV rep gene expressing Rep protein which functions as integrase; another plasmid, pTRP5GFPFVIII-BDD, contains B-domain-deleted FVIII (FVIII-BDD) and GFP gene flanked by AAV ITRs. AAV ITRs contains cis elements recognized by Rep protein and guides the site specific integration event. Human embryonic kidney (HEK) 293 cells were co-transfected with the two plasmids and resulted cells were screened by green fluorescence to establish the recombinant FVIII-BDD cell line. The cell line produced active FVIII-BDD that was secreted into the cultured media. The cell line development process took 3 months.

## Materials and methods

### Construction of the recombinant cell line

Standard procedures were followed for plasmid construction, growth, and purification. The plasmid pZp9FVIII△BS containing the B-domain deleted FVIII-BDD cDNA was used as the template for polymerase chain reaction (PCR) (a gift from Prof. Cheng-Wu Chi, Shanghai Institute of Biochemistry, Academia Sinica, Shanghai, China). pTRP5GDNFGFP and pSVAV2 were kindly provided by Dr. Berns, University of Florida, US. The GDNF gene of the plasmid pTRP5GDNFGFP was replaced with FVIII-BDD gene and the resultant plasmid was named as pTRP5GFPFVIII-BDD.

The human embryonic kidney 293 cell line was obtained from the Cell Bank of the Chinese Academy of Sciences (Shanghai, China). Monolayer cultures of HEK 293 cells were maintained in Dulbecco’s modified Eagle’s medium (DMEM) (Hyclone, USA) supplemented with 10% fetal bovine serum (Gibco, USA) and 1% penicillin and streptomycin (Beyotime, China). HEK 293 cells (1 × 10^5^) were seeded in 24-well plates and co-transfected with pTRP5GFPFVIII-BDD and pSVAV2 at ratio of 50: 1 using Polyethylenimine (PEI) according to the manufacturer’s instructions. 25 kDa linear PEI, were purchased from Polyscience (Warrington, PA), and used to prepare a stock 50 mM solution. Then we set up two tubes with either 1 μL of pTRP5GFPFVIII-BDD and 20 ng of pSVAV2, or 0.9 μL of PEI, added 50 μL of HBS (20 mM Hepes buffer, pH 7.4, 150 mM NaCl) to each and incubated them at room temperature for 10 min. The two dilutions were then mixed together and incubated at room temperature for an additional 10 min to allow the formation of PEI-DNA complexes. The reaction mixture was added to the monolayers. After 3 days of transfection, cells were subcultured twice by diluting 1:3. Cells were cloned as follows: cells were plated at 10 cells per well into 96-well plates. Two weeks later, wells containing cells expressing green fluorescence were identified and expanded into a 24-well plate, grown for 5 days, and subsequently cloned into 96-well plate at one cell per well, and the above process was repeated. The single cell cloned fluorescence-positive cell clones were enlarged. The percentage of GFP-positive cells of a selected clone was examined by flow cytometric analysis (FACS, BD LSRFortessa™).

### PCR analysis of the site-specific integration of FVIII-BDD gene

Total genomic DNA was isolated from GFP expression HEK 293 cells. The transgenes were examined by PCR performed with FVIII primers (sense primer: 5′-CATCGCTAGCGCCACCATGCAAATAGAGCTC, anti-sense primer: 5′-GCAGAACCAATGCATTCAGTAGAGGTCCTG), and GFP primers (sense primer: 5′- AGGGGGAGGTGTGGGAGGTTTT, anti-sense primer: 5′- CCCAGCAGCGGTCACAAACT), respectively. The site-specific integration was confirmed with AAV D-sequence sense primer HAIJP1 (5′-AGGAACCCCTAGTGATGGAG) and a human chromosome 19 AAVS1 -specific anti-sense primer HAIJP2 (5′-TCAGAGGACATCACGTGGTG) [16].

### FVIII-BDD expression analysis

Total RNA was extracted from recombinant FVIII-BDD cell line using Trizol Reagent (Sigma, USA) following the manufacturer's instructions. RNA (500ng) was reverse transcribed to cDNA with oligo (dT) primer according to the instruction of RevertAid First Strand cDNA Synthesis Kit (Thermo, USA). Real-time PCR assay was applied to determine the expression of FVIII-BDD with cDNA (1 μL cDNA diluted 1:10). Gene expression of FVIII-BDD was quantified using the SYBR Green PCR Master Mix kit (Tiangen, Beijing) and FVIII specific primers (sense primer: 5′ TGATGATGACAACTCTCCTT, anti-sense primer: 5′ TCTTCAGCAGCAATGTAATG) on the 7500 Real-Time PCR System (Applied Biosystems). Cycling was carried out for 2 min at 50°C, followed by denaturation at 95°C for 10 min. Amplification was carried out with 40 cycles of 95°C for 15 s and at 60°C for 1min. The specificity of each primer pair was confirmed by melting curve analysis. The GAPDH gene was used as an endogenous control (housekeeping gene). All reactions were carried out in triplicate, and the relative expression levels were calculated using the comparative CT method with the mean of the virgin cells used as reference [17].

The cloned cells were cultured on T-25 flask; the media was changed to serum-free media when they reached a confluence of above 90%. Following cultured in serum-free media for 48h, media were collected and the total proteins in media were concentrated by using Amicon® Ultra-4 Centrifugal Filter Devices (Millipore, USA) and resolved on 8% SDS-PAGE, and probed with Factor VIII (S2194) pAb (Bioword, USA). Unbound antibodies were washed from the blots with Tris-Tween buffered saline (TBST) solution and subsequently the membrane was incubated with an alkaline phosphatase-conjugated polyclonal goat anti-rabbit secondary antibodies (Abgent, USA) at 1: 5,000 dilution. After removing the secondary antibodies with wash buffer, blots were treated with chemiluminescent substrate (Millipore, USA). The blot was then exposed to X-ray film. Untransfected cells following the same experimental procedure were used as negative controls.

### Procoagulant activity assay of FVIII-BDD

FVIII-BDD protein activity was measured by the chromogenic-based method using the Coamatic factor VIII (Chromogenix, Italy) according to the manufacturer's instructions. The cloned cells were cultured in 24-well plate; serum-free and phenol red free DMEM medium was used when they reached a confluence of about 80%. After cultured at 37°C for 72 hours, the conditioned media was collected to measure the procoagulant activity of FVIII-BDD. The standard curve 0-1 IU/mL was prepared using a recombinant coagulation factor VIII (Bayer, Germany), and HEK 293 cells were used as the control.

## Results

**Figure 1:**
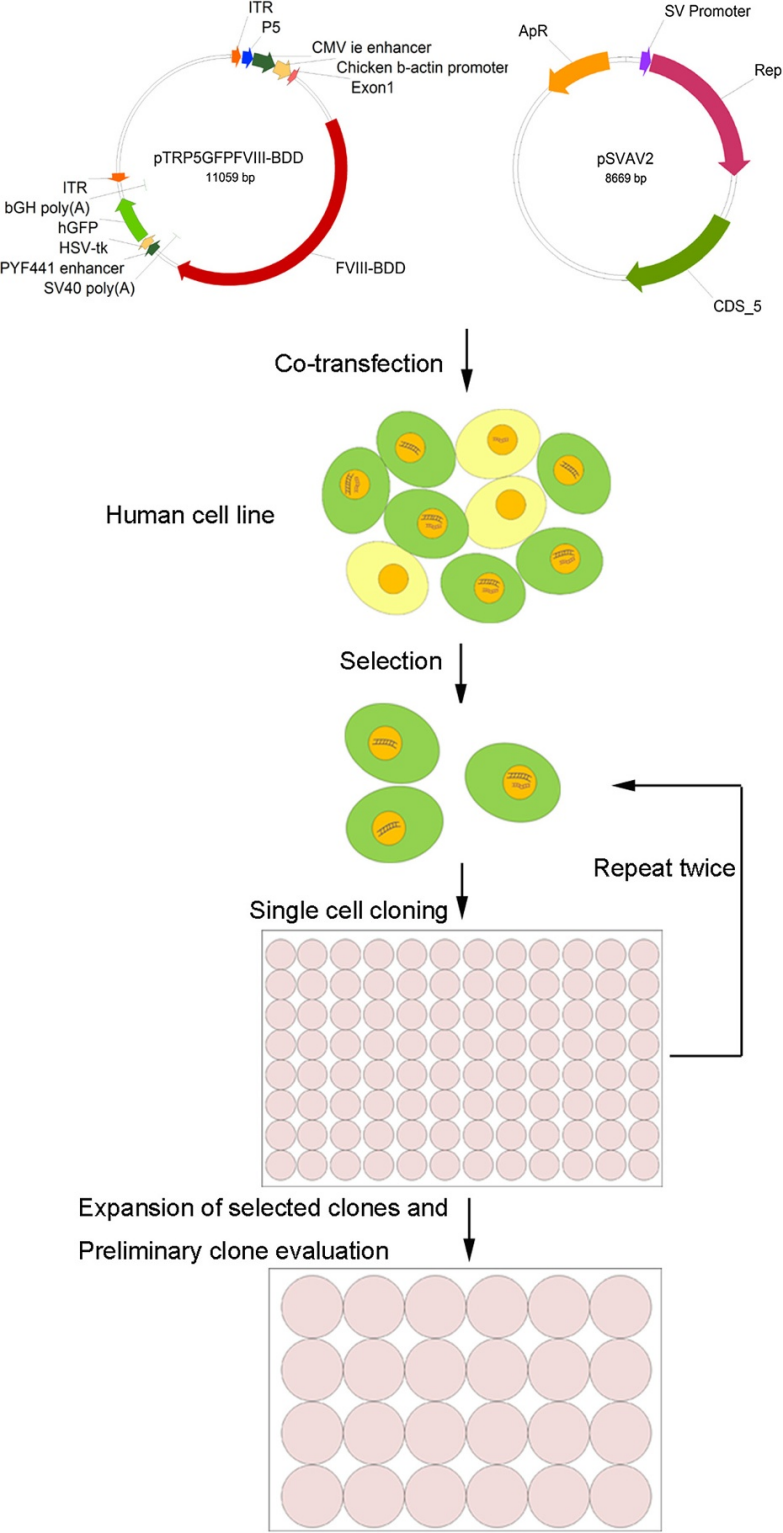
**Illustration of a typical process to develop site-specific integration of transgene expressing human cell lines.** In this process, two plasmids are essential. One plasmid contains the gene of interest and GFP gene with associated regulatory elements flanked by two copies of the AAV ITR. This construct contains the cis-acting IEE sequence from the P5 promoter, which is required for optimal site-specific integration. Another plasmid contains AAV rep gene providing an integration enzyme. HEK 293 cells were co-transfected with the two plasmids at ratio of 50: 1 using PEI. Transfected cells are subcloned into 96-well plates three times and screened by green fluorescence to establish the GFP expression human cell lines. The fluorescence-positive single cell clones were chosen for progressive expansions and further clone evaluations.

**Figure 2:**
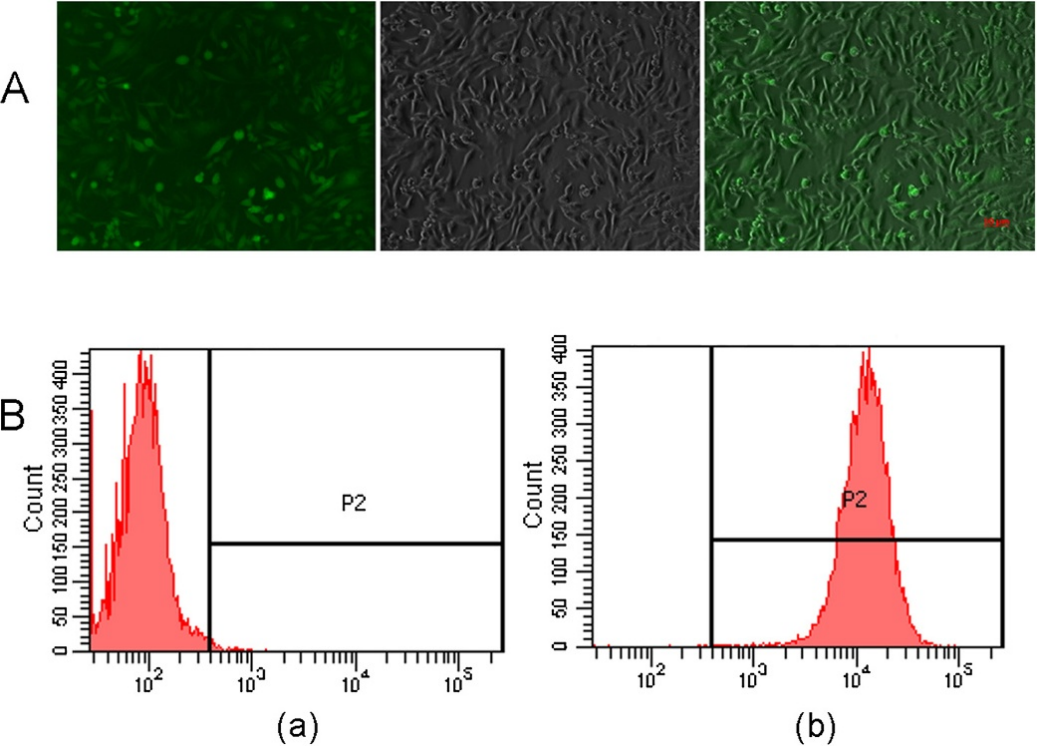
**GFP expression of a selected stable cell clone of pTRP5GFPFVIII-BDD transduced HEK 293 cell. A)**. Green fluorescent microscope picture of the pTRP5GFPFVIII-BDD transduced stable HEK 293 cell line. Scale bar corresponds to 10 μm. **B)**. Flow cytometric analysis of GFP expression of a selected stable cell clone of pTRP5GFPFVIII-BDD transduced HEK 293 cell. **(a)** Negative HEK 293 cell; **(b)** The cloned HEK 293 cell line, showing that 99.9% were GFP positive.

For the site specific integration of AAV into human chromosome 19, we used two plasmids, a Rep expression plasmid and a transgene integration plasmid. The transgene integration vector pTRP5GFPFVIII-BDD contains the FVIII-BDD gene, which is driven by the cytomegalovirus (CMV) enhancer and chicken β-actin (CBA) promoter, and the GFP gene expression were driven by the herpes simplex thymidine kinase promoter. Gene cassettes were flanked between AAV ITRs. The AAV ITR contains cis-acting IEE sequence from the P5 promoter, which is required for optimal site-specific integration (Figure 1). Human embryonic kidney (HEK) 293 cells were co-transfected with the two plasmids and screened by green fluorescence. After 3 days of transfection, cells were subcultured twice. When the cells divided, the vectors unintegrated were diluted out. Followed by 3 times of single cell cloning, six GFP-expression cell clones were obtained (Figure 2A). Flow cytometric analysis indicated that the percentage of GFP-positive cells of a selected clone was 99.9% (Figure 2B).

PCR analysis was employed to detect whether FVIII-BDD gene has been integrated into the AAVS1 site of the GFP-expression cells. First, the FVIII-BDD primers and GFP primers were used to test the complete integration of the FVIII-BDD gene.

**Figure 3:**
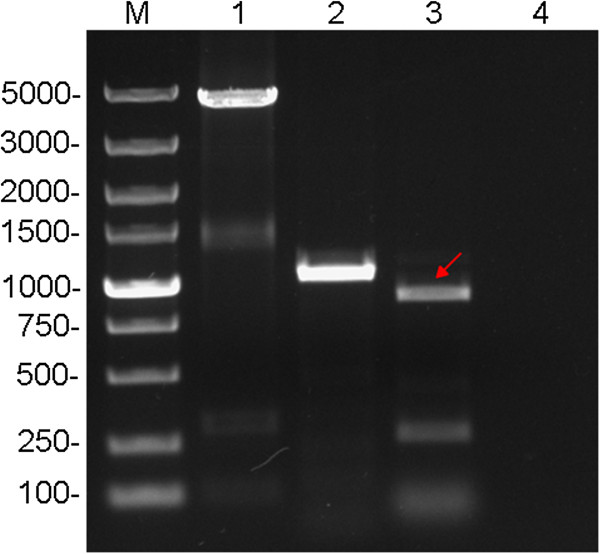
**PCR analysis of transgene in the cloned cell line genome.** Lane 1. FVIII-BDD gene; Lane 2. GFP gene; Lane 3. AAVS1 site-specific integration, the red arrow indicated the product from junction PCR (amplification with AAV D-sequence primer and an AAVS1-specific primer); Lane 4. Negative control.

The results showed a 4.4 kb product indicating the full length of FVIII-BDD gene (Figure 3, lane 1) and a 1 kb product indicating partial of GFP gene cassette (Figure 3, lane 2). The subsequent sequencing analysis demonstrated that the FVIII-BDD gene has no mutation. Then, we used junction PCR assay to further investigate whether the FVIII-BDD integrated at AAVS1 site or not. A rAAV sense-primer, HAIJP1, and an AAVS1 anti-sense-primer, HAIJP2, were used to carry out the PCR (Figure 3, lane 3). The results indicated that FVIII-BDD gene was integrated into AAVS1 site of chromosome 19. The FVIII-BDD site specific integrated HEK 293 cell line was established and named as HEK 293-FVIII-BDD.

**Figure 4:**
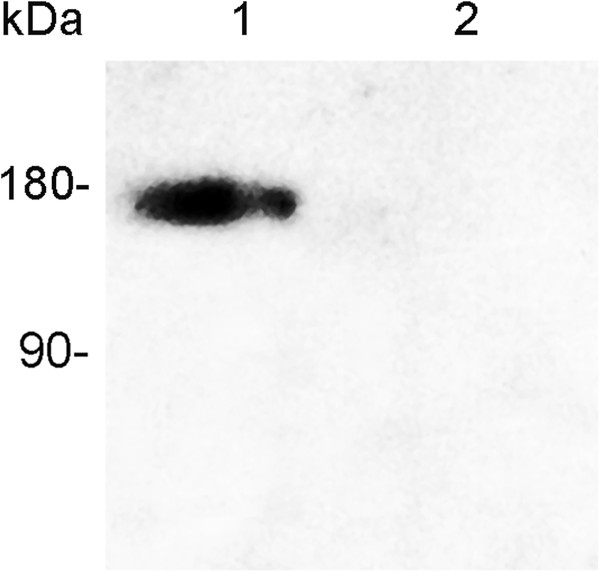
**FVIII-BDD protein expression in the cloned cell lines.** The total proteins in the cultured serum-free media of cloned cells were concentrated to identify the expression of FVIII-BDD proteins, and probed with Factor VIII (S2194) pAb. Lane 1. HEK 293-FVIII-BDD cells; Lane 2. Mock HEK 293 cells.

We compared the expression levels of FVIII-BDD and GFP mRNAs in HEK 293-FVIII-BDD and virgin HEK 293 cells. The real-time PCR results showed that in HEK 293-FVIII-BDD cells, the amount of FVIII-BDD and GFP mRNAs were 2,236- and 3,043- fold higher than that in virgin cells. The recombinant FVIII-BDD protein in the HEK 293-FVIII-BDD cell cultured media was analysed by using western blot. A single protein band of approximately 170 kDa was identified in the media of cloned cells (Figure 4, lane 1), but not in HEK 293 cells (Figure 4, lane 2). These data showed that the FVIII-BDD gene was expressed in HEK 293-FVIII-BDD cell lines and secreted into the extracellular media.

To further characterize the function of the recombinant FVIII-BDD protein, the coagulation activity of the secreted FVIII was measured using a chromogenic assay. The cloned cells were cultured in 24-well plate, and the media were changed to serum-free and phenol red absent DMEM media when the cells reached a confluence of about 80%. The standard curve of 0-1 IU/mL was prepared using a recombinant coagulation factor VIII (Bayer, Germany). After cultured for 72 hours, the conditioned media were collected to measure the procoagulant activity of FVIII-BDD and exhibited FVIII-BDD activity of 14mU/mL.

To determine whether expression of transgene was stable over time, we maintained HEK 293-FVIII-BDD cells for 2 months to examine GFP expression by fluorescence microscopy. The cell line stably expressed GFP over 20 passages, and stably expressed FVIII-BDD was confirmed by qPCR.

## Conclusions

In the present study, we used two plasmids containing AAV ITRs and AAV rep gene to rapidly establish a stable human cell line with site specific integration of the transgene (FVIII-BDD) at AAVS1 site of human chromosome 19. The single cell cloned HEK 293-FVIII-BDD cell line consistently and stably expressed FVIII-BDD. Methodologically, we successfully developed a dual plasmids co-transfection method for the rapid establishment of a protein expression human cell line with transgene site-specific integration in human chromosome 19. This method could be used to easily and quickly produce transgene site-specific integration human cell lines for all other recombinant protein production.

## Abbreviations

rFVIII: Recombinant factor VIII
rhFVIII: Recombinant human factor VIII
FVIII-BDD: B-domain-deleted FVIII
AAV2: Adeno-associated virus type 2
ITR: Inverted terminal repeat
HEK 293: Human embryonic kidney293.
